# Histomorphological Renal Changes in the Offspring of Streptozotocin-Induced Diabetic Wistar Rats

**DOI:** 10.7759/cureus.117794

**Published:** 2026-10-12

**Authors:** Atula Imchen, Amitav Sarma, Tsusennaro Imchen, Biswajit Dey, Bishwajeet Saikia, Sarah Ralte, Pranjal P Saikia

**Affiliations:** 1 Anatomy, North Eastern Indira Gandhi Regional Institute of Health and Medical Sciences, Shillong, IND; 2 Community Medicine, Nagaland Institute of Medical Sciences and Research, Kohima, IND; 3 Pathology, North Eastern Indira Gandhi Regional Institute of Health and Medical Sciences, Shillong, IND; 4 Pharmacology (Animal House), North Eastern Indira Gandhi Regional Institute of Health and Medical Sciences, Shillong, IND

**Keywords:** hyperglycemia, kidney, maternal diabetes mellitus, offspring, renal histopathology, streptozotocin

## Abstract

Introduction: Maternal diabetes mellitus can adversely affect fetal development and may predispose offspring to metabolic and renal abnormalities. However, early renal structural changes following intrauterine exposure to maternal hyperglycemia remain incompletely characterized. This study evaluated the metabolic and histomorphological effects of maternal diabetes on rat offspring kidneys during the early postnatal period.

Methods: This experimental study was conducted using Wistar rats. Diabetes was induced in female rats using streptozotocin before mating. We evaluated offspring of diabetic and nondiabetic mothers during the first and second postnatal weeks. We assessed blood glucose levels and relative kidney weights. Kidney tissues were processed routinely for histopathology. We used hematoxylin and eosin, Masson's trichrome, and periodic acid-Schiff stains to evaluate histopathological changes. Renal morphometric parameters, including glomerular diameter and Bowman's space, were assessed using computer-assisted digital morphometry.

Results: Offspring of diabetic mothers showed significantly higher blood glucose levels during both the first and second postnatal weeks compared with controls. Relative kidney weight was also higher in the test group at both time points. Histopathological examination demonstrated focal interstitial lymphocytic infiltration in the cortex and medulla during the first postnatal week, which became more prominent during the second week. Mild cortical tubular atrophy and early interstitial fibrosis with minimal collagen deposition were observed during the second week. Morphometric analysis showed significantly increased glomerular diameter and reduced Bowman's space in the test group compared with controls during both postnatal weeks (p < 0.001).

Conclusion: Maternal diabetes was associated with persistent hyperglycemia, increased relative kidney weight, and early structural alterations in the kidneys of rat offspring. The progressive inflammatory, tubular, fibrotic, and glomerular changes observed during the early postnatal period suggest that maternal hyperglycemia may adversely influence renal development and potentially increase susceptibility to later-life renal complications.

## Introduction

Diabetes mellitus is a group of metabolic disorders characterized by hyperglycemia resulting from defects in insulin secretion, insulin action, or receptor nonresponsiveness [1]. Chronic hyperglycemia causes progressive damage to multiple organ systems, particularly the cardiovascular, renal, nervous, and ocular systems [2]. The kidneys are especially susceptible because of their role in glucose filtration and their sensitivity to microvascular injury.

Diabetic nephropathy, a major cause of chronic kidney disease (CKD), affects both type 1 and type 2 diabetes. Approximately 50% of individuals with type 2 diabetes and one-third of those with type 1 diabetes develop CKD [3,4]. Maternal hyperglycemia has significant adverse effects on fetal development. Persistent hyperglycemia is the hallmark of diabetes mellitus and is associated with metabolic disturbances that contribute to the development of chronic complications [5]. A previous study from the present institute by Sarma et al. demonstrated that adverse intrauterine exposure can affect placental function and fetal development, highlighting the potential impact of the intrauterine environment on offspring health [6]. Experimental and epidemiological studies have also shown that infants born to mothers with preexisting diabetes have a higher risk of congenital anomalies than those born to nondiabetic mothers [7]. In addition, exposure to intrauterine hyperglycemia increases the risk of obesity and metabolic syndrome later in life, highlighting the long-term impact of maternal diabetes on offspring health [8].

Animal models, particularly rodents, are widely used to investigate the developmental effects of maternal diabetes. Streptozotocin (STZ)-induced diabetes is a well-established model that selectively destroys pancreatic β-cells, resulting in sustained hyperglycemia similar to human diabetes [9,10]. This model is commonly used to study the effects of maternal hyperglycemia on fetal and postnatal development.

Although the systemic effects of maternal diabetes have been extensively studied, information on the histological changes in the kidneys of offspring exposed to intrauterine hyperglycemia remains limited. Understanding these structural alterations may provide insight into the early renal changes that predispose offspring to future kidney dysfunction.

Therefore, the present study aimed to evaluate gross morphology, blood glucose levels, and histomorphological changes in the kidneys of offspring born to STZ-induced diabetic Wistar rats during the first and second postnatal weeks. The findings may improve our understanding of the early renal effects of maternal hyperglycemia and their potential implications for later renal function.

## Materials and methods

Study design

This experimental study was conducted over 15 months (October 2023 to January 2025) using healthy adult female Wistar rats (*Rattus norvegicus*). Previously unmated female rats weighing 175-200 g were procured from the Animal House, Department of Pharmacology. Animals were acclimatized for one week in polypropylene cages with sterilized paddy husk bedding under controlled conditions (23°C ± 2°C, 55% ± 5% relative humidity, 12-hour light/dark cycle) with free access to a standard pellet diet and water. Animals with systemic illness were excluded.

Ten female rats were used in each of the control and diabetic groups to minimize animal use. After confirming baseline fasting blood glucose levels below 150 mg/dL, the animals were randomly assigned by lottery to the respective groups. Diabetes was induced in the test group by a single intraperitoneal injection of STZ (65 mg/kg; Otto Chemie, India) dissolved in citrate buffer (pH 4.5) following a six- to eight-hour fast. To prevent postinjection hypoglycemia, rats received 10% glucose solution for 24 hours. Ten days later, after a 6-8-hour fast, we measured fasting blood glucose from retro-orbital blood samples using an Accu-Chek glucometer (Roche Diabetes Care, Inc., Switzerland). All 10 STZ-treated rats had fasting blood glucose levels >150 mg/dL and were therefore considered diabetic and selected for breeding [10].

Each female was housed with two male rats for mating. The presence of a vaginal plug was considered confirmation of successful mating and designated gestational day 1. Pregnancy proceeded normally, and offspring were delivered after a gestation period of 21 ± 2 days. Maternal blood glucose levels were not serially monitored during pregnancy.

Ten offspring from each maternal group were randomly selected by lottery. In both the control and diabetic groups, five offspring were sacrificed during the first postnatal week and five during the second postnatal week. Animals were deeply anesthetized before intracardiac blood collection for glucose estimation using an Accu-Chek glucometer. As the timing of blood collection relative to feeding was not standardized, the glucose measurements were considered random blood glucose measurements. The kidneys were removed, weighed, and examined separately. Kidney tissues were processed routinely for histopathology and stained with hematoxylin and eosin, Masson's trichrome (MT), and periodic acid-Schiff (PAS) for histopathological evaluation. Histopathological assessment was performed by a pathologist blinded to the maternal group allocation.

High-resolution digital photomicrographs (×40 objective) of all kidney sections were captured and analyzed using computer-assisted morphometric software (CellSens - Olympus, Olympus Corporation, Japan). Glomerular diameter and Bowman's space were measured in five randomly selected microscopic fields per tissue section, with five measurements per field, yielding 25 measurements per section. The mean value for each parameter was used for statistical analysis.

Study participants

The study population comprised the offspring of control and STZ-induced diabetic female Wistar rats. Ten offspring from each maternal group were randomly selected, with five offspring evaluated during the first postnatal week and five during the second postnatal week.

Ethical considerations

The study was approved by the Institutional Animal Ethics Committee (Approval No. NEIGR/Pharma-AH/IAEC-Part-11/2023/47).

Statistical analysis

Data were analyzed using IBM Statistical Package for the Social Sciences Statistics version 21 (IBM Corp., Armonk, NY). Normality was assessed using the Shapiro-Wilk test. Continuous variables are presented as mean ± standard deviation. Differences between the control and test groups at each postnatal time point were assessed using the independent-samples Student's t-test. A two-tailed p value of <0.05 was considered statistically significant.

## Results

Mean blood glucose levels were significantly higher in the offspring of the test group than in the control group at both postnatal time points. During the first postnatal week, mean blood glucose levels were 139 ± 8.14 mg/dL in the test group and 112 ± 14.21 mg/dL in the control group (p = 0.006). During the second postnatal week, the corresponding values were 156.2 ± 18.38 and 120 ± 10.02 mg/dL, respectively (p = 0.005).

Relative kidney weights were higher in the offspring of diabetic mothers than in controls during both postnatal weeks; however, the differences were not statistically significant. For the right kidney, the mean relative weight was 0.670% ± 0.044% in the test group vs. 0.624% ± 0.062% in controls during the first postnatal week (p = 0.211), and 0.676% ± 0.064% vs. 0.626% ± 0.023% during the second week (p = 0.140). For the left kidney, the corresponding values were 0.672% ± 0.071% vs. 0.590% ± 0.058% (p = 0.081) and 0.680% ± 0.061% vs. 0.620% ± 0.023% (p = 0.074), respectively.

The kidneys of the control group's offspring showed preserved renal architecture during the first postnatal week, with a well-defined cortex and medulla. The renal corpuscles exhibited well-developed glomerular tufts enclosed by Bowman's capsules, with distinct urinary spaces. The proximal and distal convoluted tubules were lined by intact simple cuboidal epithelium, while the loops of Henle and collecting ducts appeared unremarkable (Figure 1A). Similarly, the kidneys of the test group maintained preserved renal architecture. The renal corpuscles, proximal and distal convoluted tubules, loops of Henle, and collecting ducts were well preserved, with no evidence of tubular atrophy, necrosis, or interstitial fibrosis. The only notable finding was focal interstitial lymphocytic infiltration in both the renal cortex and medulla. During the second postnatal week, the control group continued to exhibit preserved renal architecture with normal glomeruli, Bowman's capsules, proximal and distal convoluted tubules, loops of Henle, and collecting ducts, without significant histopathological abnormalities (Figure 1B).

**Figure 1:**
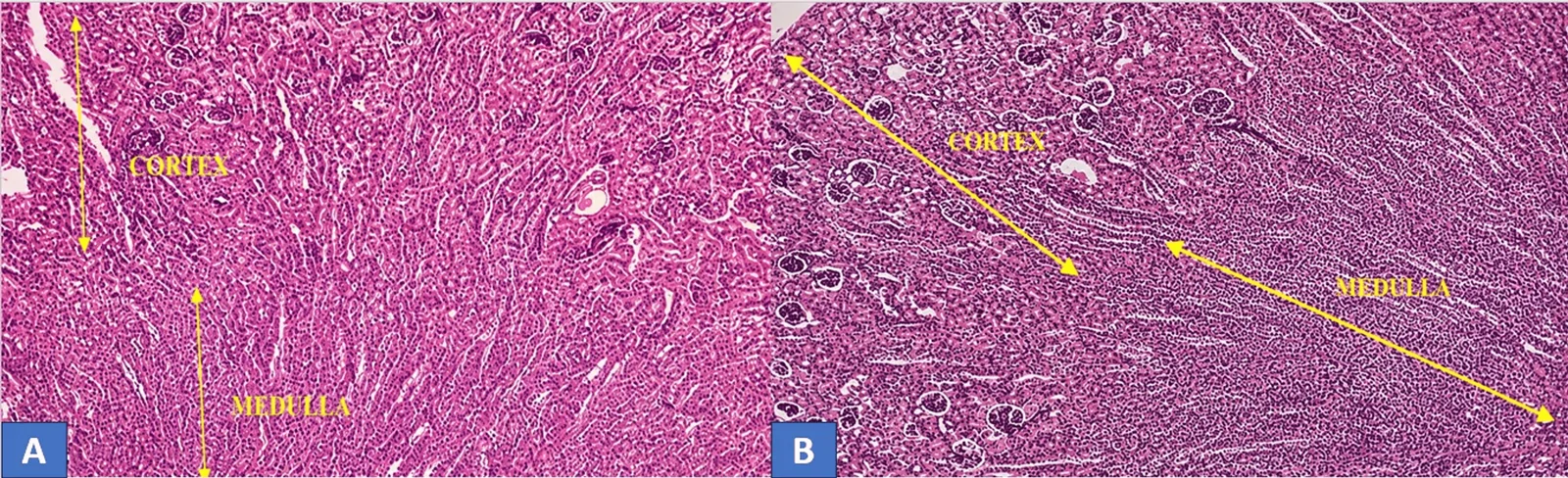
Histological sections of the kidney showing normal renal architecture with well-defined cortex and medulla in the control group during the (A) first and (B) second postnatal weeks (H&E, ×10) H&E: hematoxylin and eosin

In contrast, the test group demonstrated mild histopathological alterations. There was increased interstitial lymphocytic infiltration involving both the renal cortex and medulla (Figure 2A). Mild tubular atrophy, characterized by reduced tubular diameter and epithelial cell size, was observed in some cortical tubules with no evidence of tubular necrosis (Figure 2B). MT staining demonstrated mild interstitial fibrosis with minimal collagen deposition in the renal interstitium (Figure 2C).

**Figure 2:**
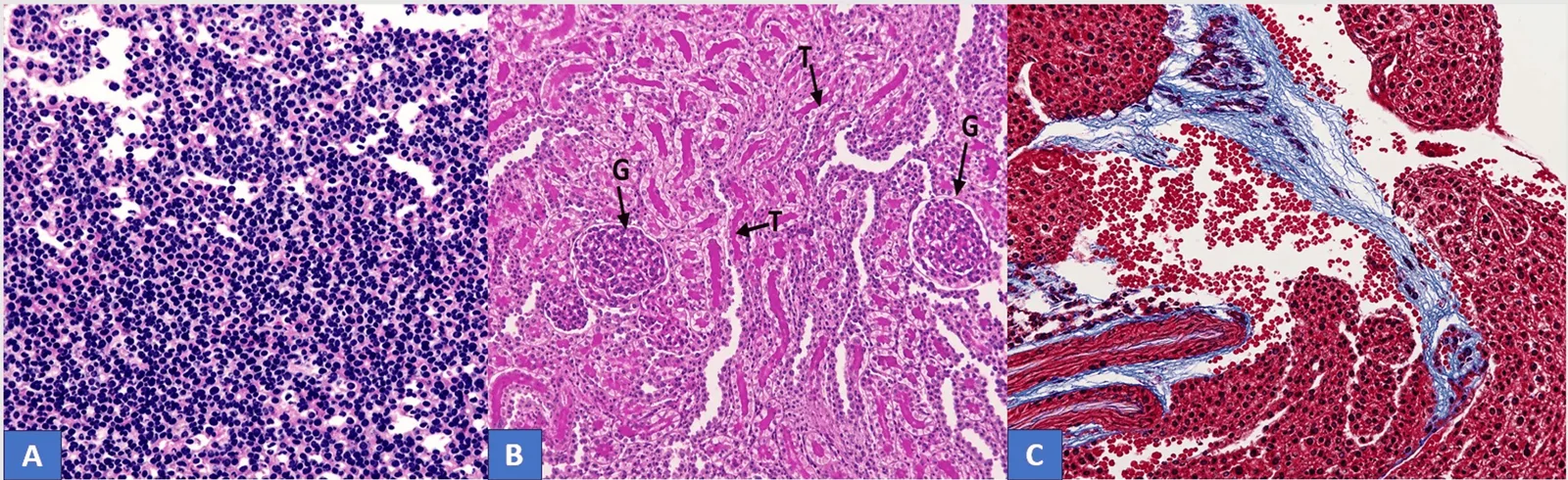
Histological changes in the kidneys of the test group during the second postnatal week. (A) Interstitial lymphocytic infiltration in the renal parenchyma (H&E, ×40). (B) Enlarged glomeruli (G) and tubules (T) of varying sizes (PAS, ×40). (C) Mild interstitial fibrosis with collagen deposition (Masson’s trichrome, ×40) H&E: hematoxylin and eosin; PAS: periodic acid-Schiff

Morphometric analysis revealed marked glomerular hypertrophy and narrowing of Bowman's space in the offspring of diabetic mothers during both postnatal weeks (Table 1). In the first postnatal week, the right and left glomerular diameters in the test group measured 34.8 ± 0.84 and 34.4 ± 0.55 µm, respectively, compared with 19.6 ± 0.55 and 19.4 ± 0.55 µm in the control group (both p < 0.001). Conversely, Bowman's space was reduced to 1.8 ± 0.84 µm (right) and 2.0 ± 1.0 µm (left) in the test group, compared with 8.2 ± 0.45 and 8.4 ± 0.55 µm, respectively, in the control group (both p < 0.001). A similar pattern was observed during the second postnatal week, with glomerular diameters increasing to 37.8 ± 0.84 µm (right) and 36.8 ± 0.84 µm (left), while Bowman's space remained reduced at 3.2 ± 0.84 and 3.0 ± 0.71 µm, respectively (all p < 0.001).

**Table 1:** Comparison of renal morphometric parameters between control and test groups during the first and second postnatal weeks Values are presented as mean ± SD. An independent-samples Student’s t-test was used for between-group comparisons at each postnatal time point. A two-tailed p < 0.05 was considered statistically significant SD: standard deviation

Renal morphometric parameter	Control (first week)	Test (first week)	p value	t-statistic	Control (second week)	Test (second week)	t-statistic	p value
Right Bowman's space (µm)	8.20 ± 0.45	1.80 ± 0.84	<0.001	15.018	15.00 ± 0.71	3.20 ± 0.84	23.990	<0.001
Right glomerular diameter (µm)	19.60 ± 0.55	34.80 ± 0.84	<0.001	-33.851	22.00 ± 0.71	37.80 ± 0.84	-32.122	<0.001
Left Bowman's space (µm)	8.40 ± 0.55	2.00 ± 1.00	<0.001	12.539	14.40 ± 0.55	3.00 ± 0.71	28.383	<0.001
Left glomerular diameter (µm)	19.40 ± 0.55	34.40 ± 0.55	<0.001	-43.122	21.80 ± 0.84	36.80 ± 0.84	-28.235	<0.001

## Discussion

The present study evaluated the metabolic and structural effects of maternal diabetes on the kidneys of neonatal rat offspring during the first two postnatal weeks. Offspring of diabetic mothers exhibited significantly higher blood glucose levels than controls at both postnatal time points, indicating persistent metabolic dysregulation during early postnatal life. These findings were consistent with those of Aref et al., who similarly reported elevated fasting serum glucose levels in the offspring of diabetic mothers during the first two postnatal weeks, supporting the concept that intrauterine hyperglycemia was associated with early and sustained metabolic alterations [11].

A trend toward higher relative kidney weight was observed in the offspring of diabetic mothers during both postnatal weeks, although the differences did not reach statistical significance. This trend may indicate early renal growth or hypertrophic adaptation associated with maternal diabetes. These findings were comparable with those of Tran et al., who demonstrated that early-life glycemic stress altered renal morphogenesis and promoted renal enlargement [12]. Such an increase in kidney weight may reflect adaptive responses to hyperglycemia, including compensatory glomerular hyperfiltration and increased metabolic workload, which are recognized early events in the pathogenesis of diabetic nephropathy [13].

Nephrogenesis in rats begins around gestational day 11 and continues until approximately postnatal days 11-15, making this period particularly vulnerable to maternal metabolic disturbances [14]. In the present study, focal interstitial lymphocytic infiltration was observed in both the renal cortex and medulla during the first postnatal week, which became more pronounced during the second week. Similar inflammatory changes had been reported by Zafar et al., suggesting that maternal hyperglycemia initiated an early inflammatory response in the developing kidney [15].

In addition to inflammatory cell infiltration, mild tubular atrophy and early interstitial fibrosis were observed during the second postnatal week. These findings were consistent with previous experimental studies demonstrating renal structural alterations, including tubulointerstitial injury and fibrosis, in offspring exposed to maternal diabetes [16]. Although the histological changes observed in the present study were relatively mild, they may represent the initial stages of renal remodeling that precede overt diabetic nephropathy.

Morphometric analysis revealed notable glomerular hypertrophy and a reduction in Bowman's space in the offspring of diabetic mothers. Similar enlargement of the glomerular area had been reported by Magaton et al. in offspring of STZ-induced diabetic Wistar rats [17]. Glomerular hypertrophy is considered an early adaptive response to increased intraglomerular pressure and hyperfiltration. Intrauterine hyperglycemia may impair nephrogenesis, resulting in a reduced nephron endowment. Consequently, the remaining nephrons undergo compensatory hypertrophy to accommodate the increased functional demand, thereby predisposing the kidney to progressive structural injury later in life.

In contrast, Teoh et al. reported reduced kidney size with more advanced pathological changes, including glomerular degeneration, inflammatory cell infiltration, and basement membrane thickening, in untreated diabetic animals examined on the 10th day [18]. These severe alterations were not observed in the present study, possibly because the offspring were evaluated during the early postnatal period, when renal injury was still evolving, and irreversible structural damage had not yet developed.

The severity of renal injury in offspring was likely influenced by both the degree and duration of maternal hyperglycemia. Experimental evidence suggested that exposure to intrauterine hyperglycemia disrupted nephrogenesis, induced oxidative stress, and activated molecular pathways that promoted inflammation, fibrosis, and epigenetic modifications, ultimately increasing susceptibility to hypertension and CKD in later life [12,19]. These findings underscored the importance of optimal glycemic control during pregnancy to minimize adverse renal programming and reduce the long-term risk of kidney disease in the offspring.

The study was limited by the short follow-up period, which was restricted to the first two postnatal weeks. Maternal blood glucose levels were not serially monitored during pregnancy; therefore, the degree and duration of maternal hyperglycemia throughout gestation could not be assessed. Potential litter effect could not be assessed or excluded, and pseudoreplication related to litter clustering could not be definitively ruled out. Offspring sex and body-weight data were not incorporated into the analysis. Histological changes were evaluated qualitatively rather than using a standardized quantitative scoring system. Renal function was not assessed using biochemical markers, and the molecular mechanisms underlying the observed histopathological changes were not investigated.

## Conclusions

This study demonstrates that maternal diabetes induces early metabolic and renal alterations in the offspring of STZ-induced diabetic Wistar rats during the first two postnatal weeks. Maternal hyperglycemia was associated with persistent hyperglycemia in the offspring, increased relative kidney weight, glomerular hypertrophy, inflammatory cell infiltration, and early fibrotic and tubular changes. Although the renal lesions were mild, their progression between the first and second postnatal weeks suggested that intrauterine exposure to maternal diabetes may have initiated early renal remodeling, warranting further investigation of its potential long-term renal consequences. These findings underscore the importance of optimal maternal glycemic control during pregnancy and provide a basis for further longitudinal studies to determine whether these early renal alterations persist into adulthood.
